# Mechanisms of Mitochondrial Impairment by SARS-CoV-2 Proteins: A Nexus of Pathogenesis with Significant Biochemical and Clinical Implications

**DOI:** 10.3390/ijms26209885

**Published:** 2025-10-11

**Authors:** Marco Refrigeri, Alessandra Tola, Rosangela Mogavero, Maria Michela Pietracupa, Giulia Gionta, Roberto Scatena

**Affiliations:** 1 Clinical Pathology Department, Vannini Hospital, Via di Acqua Bullicante 4, 00177 Rome, Italy; 2 Clinical Pathology Department, Ospedale “Giovanni Paolo II”, 07026 Olbia, Italy

**Keywords:** SARS-CoV-2, interactoma, mitochondria, oxidative stress, ROS, long COVID, mitochondrial disease, oxidative phosphorylation, mitochondrial antiviral signaling (MAVS), mitophagy, mitochondrial bioenergetics, inflammasome activation (e.g., NLRP3), viral immune evasion, mitochondrial fission/fusion dynamics, mitochondrial damage-associated molecular patterns (mtDAMPs), biomarkers, diagnosis, prognosis

## Abstract

Severe acute respiratory syndrome coronavirus 2 (SARS-CoV-2) closely interacts with host cellular mechanisms, with mitochondria playing a crucial role in this process. As essential organelles that control cellular energy production, apoptosis, reactive oxygen species (ROS) metabolism, and innate immune responses, mitochondria are vital to the development of COVID-19. However, the exact molecular interactions between mitochondria and SARS-CoV-2 remain under active investigation. Gaining a comprehensive understanding of mitochondrial involvement in SARS-CoV-2 infection is therefore essential for uncovering complex disease mechanisms, identifying prognostic biomarkers, and developing effective treatments. Ultimately, exploring these virus–host interactions may provide new insights into the fundamental and complex aspects of mitochondrial physiology and pathophysiology.

## 1. Introduction

SARS-CoV-2, the virus responsible for the COVID-19 pandemic, significantly affected global health. By January 2023, official reports indicated over 664 million confirmed cases and more than 6.7 million deaths worldwide [1,2]. Since the beginning of the COVID-19 pandemic, numerous mutations of SARS-CoV-2 have been identified. Periodic viral genomic sequencing helps to detect new genetic variants circulating in communities [3]. An updated version of the SARS-CoV-2 phylogenetic tree is shared on GISAID platform (Global Initiative on Sharing Avian Influenza Data) [3].

SARS-CoV-2 is an enveloped, spherical virus that belongs to the Betacoronavirus genus. This group of single-stranded RNA viruses mainly infects mammals, with bats and rodents often serving as natural reservoirs. Other betacoronaviruses that infect humans include MERS-CoV, SARS-CoV, and viruses that cause the common cold, such as OC43 [4]. 

The virus particles have a diameter of approximately 60 to 140 nm, and these coronaviruses have some of the largest genomes among single-stranded RNA viruses. The SARS-CoV-2 genome is approximately 27 to 32 kilobases (kb) long. It is a positive-sense RNA strand, meaning the host cell’s machinery can directly translate it. The genetic sequence contains multiple open reading frames (ORFs), which are segments that code for different proteins [2,4].

The physical interactions between SARS-CoV-2 proteins and human cells are crucial for almost every stage of the viral life cycle. These interactions are significant for processes such as entering host cells, replicating the viral genome, assembling new virions, and suppressing the host’s immune response [5]. 

To clarify these molecular mechanisms, researchers have mapped the SARS-CoV-2–human interactome using high-throughput techniques such as affinity purification mass spectrometry (AP-MS) and yeast two-hybrid (Y2H) screening. These studies revealed a wide range of interactions between various SARS-CoV-2 proteins and their corresponding human targets. The resulting interactome map serves as a valuable resource for a deeper understanding of the pathogenic molecular mechanisms underlying SARS-CoV-2 infections. Moreover, this complex network of interactions is essential for discovering potential prognostic and therapeutic targets for COVID-19 [6,7,8].

This article focuses on a specific aspect of COVID-19’s pathophysiology. We explore the interactions between particular SARS-CoV-2 proteins and host mitochondria. Analyzing the structural and functional relationships between various SARS-CoV-2 proteins and mitochondria can help clarify some complex and debated mechanisms of COVID-19. Ultimately, this could have important implications for diagnosis, outcome prediction, and treatment of the infection, as well as for a broader understanding of mitochondrial physiology and pathophysiology.

### 1.1. SARS-CoV-2 Proteome and Mitochondria: Structural and Functional Relationships with Clinical Implications

To better understand the pathophysiology of how SARS-CoV-2 proteins interact with mitochondria, it is helpful to remember that the entire SARS proteome can be divided into structural proteins, non-structural proteins, and accessory proteins (Table 1) [8,9,10].

a. Structural Proteins

Four main structural proteins make up the basic structure of the virus particle (virion):

1. Spike (S) protein: This trimeric protein is located on the virus’s surface and gives coronaviruses their distinctive “crown-like” appearance. It enables the virus to enter host cells by binding to the ACE2 receptor on the cell surface.
2. Nucleocapsid (N) protein: This protein binds to the viral RNA genome to facilitate the formation of the helical ribonucleocapsid complex.
3. Membrane (M) protein: This is the most abundant structural protein and is crucial for the virus’s assembly.
4. Envelope (E) protein: This small protein helps assemble new virus particles and form the viral envelope.

b. Non-Structural Proteins

About two-thirds of the genome, beginning from the 5’ end, includes the ORF1a/b region. This segment encodes two large polyproteins (pp1a and pp1ab), which are then cleaved into 16 non-structural proteins (nsps), labeled NSP1 through NSP16. These NSPs are primarily involved in replicating and transcribing the viral genome [4,7].

c. Accessory Proteins

SARS-CoV-2 also encodes several accessory proteins, primarily designated as ORFx. They play essential roles in viral pathogenesis and in forming specific interactions with the host immune system [8,10].

### 1.2. The SARS-CoV-2-Human Protein Interactome: A Framework for Viral Pathogenesis

#### 1.2.1. The Spike (S) Protein in Mitochondria

**Figure 1:**
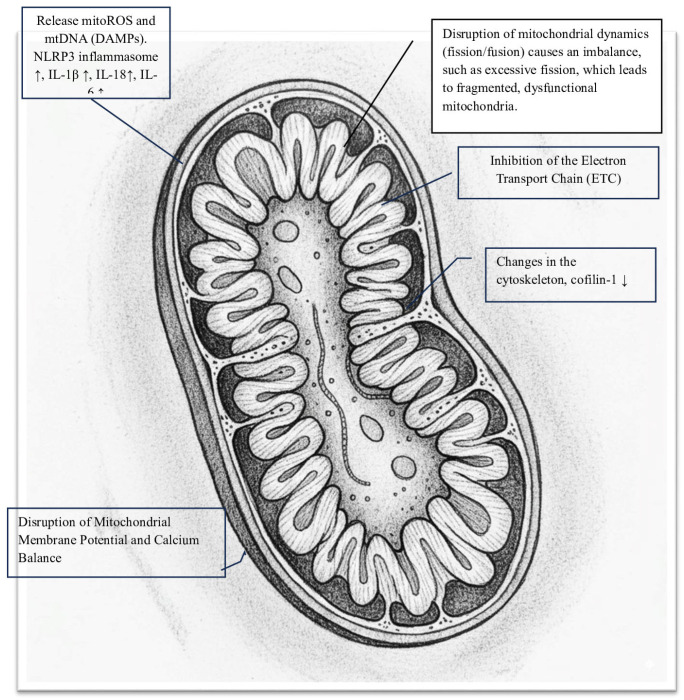
Key damaging activities of SARS-CoV-2 Spike (S) protein in mitochondria.

The SARS-CoV-2 Spike (S) protein is crucial for the virus to infect host cells. It also causes other direct and indirect harmful effects. Specifically, the Spike protein can damage cells, particularly at the mitochondrial level, triggering various harmful mechanisms that significantly impact the pathophysiology of COVID-19 (Figure 1). Notably, S protein-induced changes vary depending on the cell type (e.g., coronary artery endothelial and bronchial epithelial cells show minimal structural changes). One way it causes damage is by disrupting mitochondrial energy production [11]. Studies using recombinant S protein on isolated mitochondria or in cellular models have shown that it directly impairs mitochondrial functions, specifically:

- Inhibition of the Electron Transport Chain (ETC): The S protein significantly disrupts oxidative phosphorylation by blocking enzyme activities within the ETC, leading to a notable decrease in mitochondrial oxygen consumption rate (OCR). Spectroscopic analysis reveals that the Spike protein diminishes the intensity of heme groups in Complex III and Complex IV, suggesting a disruption of their redox functions and creating a bottleneck in electron flow [12]. Specifically, in HLMVECs, the Spike RBD markedly lowers both the basal and maximal OCRs, as well as ATP-linked respiration. In respiratory epithelial cells, exposure to the S protein results in the downregulation of key mitochondrial proteins such as SIRT3 and TOMM22, and decreases the OCR, signaling a shift away from oxidative phosphorylation [13]. This impairment of the ETC causes significant oxidative stress, as the Spike protein increases superoxide production from Complex I and Complex III. This oxidative stress further damages mitochondrial structures and functions, creating a vicious cycle [7,14,15].

Considering the indirect mechanisms of mitochondrial damage, there are:

- Cell surface receptor engagement: The interaction of the Spike protein with cell surface receptors activates signaling pathways that, along with oxidative stress, cause significant structural and functional damage to the mitochondrial network. Specifically, the engagement of the S protein’s S1 subunit with the ACE2 receptor leads to notable changes in mitochondrial shape. In human lung microvascular endothelial cells (HLMVECs), exposure to the Spike receptor-binding domain (RBD) results in mitochondrial fragmentation, swelling, and abnormal cristae remodeling, along with a decrease in cristae density [13]. Similarly, in human cardiomyocytes, prolonged exposure to the S1 subunit causes extensive mitochondrial fragmentation, confirming the role of induced mitochondrial stress. These structural damages are linked to reduced expression of TOM20, a key component of the translocase complex responsible for importing nuclear-encoded proteins into the mitochondria, indicating a disruption in mitochondrial shape and function [12].
- Disruption of mitochondrial membrane potential and calcium balance: In cardiomyocytes, mitochondrial fragmentation is associated with a loss of mitochondrial membrane potential (Δψm), increased mitochondrial calcium (mCa^2+^) levels, and heightened production of reactive oxygen species (ROS). This damage to membrane integrity and calcium imbalance triggers cell death pathways. Notably, mitochondrial dysfunction caused by the Spike protein significantly contributes to the hyperinflammation seen in severe COVID-19 cases. Damaged mitochondria release mitoROS and mitochondrial DNA (mtDNA) into the cytosol, acting as powerful danger signals known as DAMPs. These signals activate the NLRP3 inflammasome, leading to the maturation and release of pro-inflammatory cytokines IL-1β and IL-18 [9,16]. Evidence suggests that the Spike protein primes and activates the NLRP3 inflammasome in immune cells, relying on mitochondrial reactive oxygen species (mitoROS) production [17,18]. This creates a harmful cycle in which mitochondrial damage, driven by the Spike protein, promotes inflammation and systemic cellular stress. Importantly, the toxic effects of the Spike protein provide a molecular explanation for several key clinical features of COVID-19, including significant endothelial dysfunction, cardiac injury, and systemic inflammation in severe cases. Additionally, ongoing mitochondrial stress and associated metabolic changes may be key factors in the development of chronic fatigue and multi-organ symptoms in the post-acute sequelae of SARS-CoV-2 infection (PASC), underscoring the lasting impact of the Spike protein on host cell function [19,20].

#### 1.2.2. The Membrane (M) Protein in Mitochondria

The membrane (M) protein of SARS-CoV-2 is the most common structural protein of the virus. Research shows that it interacts in complex ways with the host cell’s mitochondria [21]. Although the M protein is not primarily found inside mitochondria, it can still influence their structure and functions. It does this through direct interactions with other proteins and by modifying cellular pathways, which contribute to the development of viral disease [7,20].

The M protein primarily resides in the endoplasmic reticulum (ER) and Golgi apparatus, which are essential sites for viral assembly. Its location enables it to interact with critical cellular components that link these compartments to mitochondria, especially at mitochondria-associated membranes (MAMs) [9].

On a structural level, the C-terminal endodomain of the SARS-CoV-2 M protein binds to several mitochondrial proteins. It specifically binds to the B-cell lymphoma 2 (Bcl-2) and ovarian killer (BOK) proteins, both of which are pro-apoptotic members of the Bcl-2 family. This endodomain directly interacts with the BH2 domain of BOK. This interaction binds and stabilizes BOK by preventing its ubiquitination, a process that would typically lead to its degradation [9,22].

Furthermore, the M protein interferes with the PDK1-PKB/Akt signaling pathway, which is essential for cell survival. By destabilizing this pathway, the M protein encourages apoptosis. This pro-apoptotic effect is enhanced by the SARS-CoV-2 nucleocapsid (N) protein. The N protein increases the interaction between the M protein and PDK1, amplifying the apoptotic signal [23].

Additionally, the M protein interacts with crucial parts of the mitochondrial antiviral signaling (MAVS) pathway. It binds directly to MAVS on the outer mitochondrial membrane, which is essential for the RIG-I-like receptor (RLR) signaling pathway. This pathway detects viral RNA and triggers an interferon response. The M protein’s binding to MAVS prevents the formation of a key multiprotein complex, which includes RIG-I, TRAF3, and TBK1, thereby blocking the downstream signaling needed for a strong antiviral response [5,24].

As a result, the structural interactions of the M protein with mitochondrial proteins cause three main effects on the host cell: inducing apoptosis, suppressing innate immunity, and causing mitochondrial dysfunction. The M protein also directly leads to mitochondrial dysfunction. It can induce mitochondrial fragmentation and stress signaling. These structural and functional changes result in reduced ATP production and increased reactive oxygen species (ROS), which can cause further cellular damage. This may occur because the M protein disrupts the Golgi-mitochondrial interaction, which is crucial for transporting lipids and proteins necessary for mitochondrial health. When the Golgi function is impaired, it can lead to mitochondrial problems and has been associated with neurodegenerative symptoms linked to COVID-19 [4,25].

Thus, the interaction of the SARS-CoV-2 M protein with mitochondria impairs not only mitochondrial antiviral functions but also directly inhibits mitochondrial energy production. It can sometimes trigger immune suppression and/or apoptosis. These complex disruptions underscore the M protein’s crucial role in COVID-19 pathogenesis and suggest it could be a potential target for therapeutic intervention [26].

From a clinical perspective, the pathophysiology of the SARS-CoV-2 M protein includes:

1. Neurological complications: M protein-induced mitochondrial dysfunction in neurons may cause hippocampal shrinkage, neuroinflammation, and cognitive decline. These findings support the notion that neurological symptoms associated with long COVID stem from mitochondrial derangements [5,27].
2. Pulmonary edema and ARDS: M protein damages the alveolar-capillary barrier, causing pulmonary edema, hypoxemia, and ARDS (Acute Respiratory Distress Syndrome) [28].

Finally, the M protein could serve as a target for therapies such as:

i. PI4KIIIβ inhibitors, which may reverse M-induced mitochondrial and Golgi dysfunction [29].
ii. BOK pathway inhibitors that could decrease lung injury and apoptosis [30].
iii. Antioxidants targeting mitochondrial ROS may reduce inflammation and tissue damage [31].

##### The SARS-CoV-2 Nucleocapsid (N) Protein in Mitochondria

The nucleocapsid (N) protein of SARS-CoV-2 plays a crucial role in the development of COVID-19 by directly impacting mitochondrial function. Recent research indicates that the N protein moves to the mitochondria, disrupting energy production, increasing oxidative stress, and altering the host’s immune response. This activity contributes to the inflammation observed in COVID-19 and may also be associated with long COVID [32,33].

A key aspect of the N protein’s harmful activity is its ability to localize within the host cell’s mitochondria. This localization enables it to interact with key mitochondrial components, particularly the mitochondrial antiviral-signaling (MAVS) protein. The interaction between the N protein and MAVS is crucial and can lead to the prolonged production of type I interferons (IFN), which are essential for the antiviral response. However, this prolonged activation may also lead to chronic inflammation, which is increasingly associated with the persistent symptoms of long COVID. Studies suggest that the continuous presence of the N protein, even in the absence of viral replication, contributes to creating an inflammatory environment driven by mitochondria [34].

The harmful effects of the N protein also impact essential mitochondrial functions. Research shows that the expression of the SARS-CoV-2 N protein increases reactive oxygen species (ROS) levels in mitochondria. ROS are highly reactive molecules that can further damage cells and tissues. This rise in ROS is connected to an abnormal redox state of the electron transport chain, the leading site for energy production in mitochondria. Although the N protein enhances the activity of complexes I and III in the electron transport chain, it ultimately reduces the synthesis of adenosine triphosphate (ATP), the primary energy source for cells. This disruption in energy production can impair organ function [35].

Furthermore, by interacting with MAVS, the N protein can influence the RIG-I-like receptor (RLR) signaling pathway, which is essential for detecting viral RNA and triggering an antiviral response. Some studies suggest that the N protein can inhibit the production of interferon-beta (IFN-β) stimulated by this pathway. Meanwhile, other research suggests that its interaction with MAVS may lead to a prolonged but potentially disorganized interferon response. This complex interaction shows how the N protein can disrupt the host’s immune response, thereby promoting viral replication [4,36].

The impact of the N protein on mitochondria is part of a broader viral strategy to manipulate the host’s cellular machinery. Interestingly, other SARS-CoV-2 proteins also target mitochondria, creating a redundant damage mechanism that helps the virus evade the innate immune response. This combined assault can cause severe mitochondrial dysfunction, leading to systemic inflammation and damage to multiple organs observed in severe COVID-19.

From a clinical perspective, the N protein may worsen patient outcomes by increasing host inflammation through the activation of the NF-κB and NLRP3 inflammasome pathways, especially in individuals with conditions like diabetes, cardiovascular disease, or autoimmune disorders [37].

Regarding the development of long COVID, the persistent presence of the N protein and the resulting mitochondrial dysfunction are seen as key factors in its progression. Symptoms such as fatigue and “brain fog” may be linked to the overall disruption of mitochondrial energy production [38,39].

In conclusion, the SARS-CoV-2 nucleocapsid protein localizes to and interacts with mitochondria, which is crucial for its role as a pathogen. By disrupting mitochondrial energy production, increasing oxidative stress, and altering innate immune signaling, the N protein greatly influences both the immediate inflammatory response of COVID-19 and the long-term symptoms of long COVID. Understanding these molecular mechanisms is vital for developing treatments to reduce the extensive damage caused by SARS-CoV-2 infection [34,38].

Focusing on its therapeutic and diagnostic potential, targeting N protein–MAVS interactions could help manage or lessen chronic inflammation in long COVID. Additionally, detecting N protein in cells and blood, such as peripheral blood mononuclear cells (PBMCs), might serve as a marker for ongoing viral antigenemia and autoimmune risk. Finally, its unique immunogenicity makes it a promising candidate for vaccines and serological diagnostics [38,39,40].

##### The SARS-CoV-2 Envelope (E) Protein in Mitochondria

The Envelope (E) protein is a vital membrane protein and the smallest among the four structural proteins of SARS-CoV-2. It functions as a channel-forming protein, also known as a viroporin, primarily disrupting calcium homeostasis by altering calcium signaling between the endoplasmic reticulum (ER) and mitochondria. These disruptions can cause severe cellular damage and lead to significant clinical issues [41].

The SARS-CoV-2 E protein contains 75 amino acids and has three main regions: a short N-terminal domain, a central hydrophobic transmembrane domain (TMD), and a C-terminal domain. The primary harmful effects occur at the TMD, where five E protein monomers assemble into a pentameric, flower-like structure. This configuration creates a tight, cation-selective viroporin that inserts into the membranes of the host cell’s endoplasmic reticulum (ER) and the ER-Golgi intermediate compartment (ERGIC), which are crucial sites for viral replication. It allows the free flow of various ions [41,42].

Thus, the E protein disrupts mitochondrial balance by functioning as a calcium (Ca^2+^) permeable ion channel. This harmful effect depends on the formation of pores in the ER membrane, which allows Ca^2+^ to leak from the ER and enter nearby mitochondria. This process occurs at specific contact sites, known as mitochondria-associated membranes (MAMs). The E protein induces an unregulated influx of Ca^2+^ into the mitochondria, triggering a series of damaging cellular events, including:

- Mitochondrial calcium overload: Excess Ca^2^⁺ intake by mitochondria causes the opening of the mitochondrial permeability transition pore (mPTP), which can result in cell death.
- Increased oxidative stress: This dysfunction boosts the production of mitochondrial reactive oxygen species (mtROS), which are highly damaging molecules that harm cells and impair mitochondrial function.
- Loss of mitochondrial integrity: Calcium overload and oxidative stress harm the mitochondrial membrane, leading to depolarization and triggering apoptosis (programmed cell death).
- Inflammasome activation: The E protein’s calcium transport triggers the NLRP3 inflammasome, which is essential for the innate immune response. Additionally, damaged mitochondria release mtROS and mitochondrial DNA, acting as danger signals and further promoting inflammasome activation [41,42,43].

Interestingly, the C-terminal domain has a PDZ-binding motif (PBM), which is a key virulence factor. This motif enables the E protein to interact with various host cell proteins, including ZO-1, disrupting tight junctions in epithelial cells. This disruption weakens the epithelial barrier in the airway, leading to increased inflammation and damage in the alveoli, which contributes to the development of acute respiratory distress syndrome (ARDS) [41,44].

All these activities involving mitochondria are part of SARS-CoV-2’s strategy to weaken the host cell’s defenses.

The mitochondrial dysfunction caused by the E protein has significant clinical consequences. Specifically, activation of the NLRP3 inflammasome leads to a surge of pro-inflammatory cytokines, including IL-1β and IL-18. This “cytokine storm” results in severe inflammation, causing significant tissue damage and complications in various organs, particularly in the heart and nervous system [41,45].

Notably, the effects of E protein-induced mitochondrial dysfunction seem to last beyond the acute phase of the illness. This ongoing damage might contribute to Post-Acute Sequelae of COVID-19 (PASC), also known as Long COVID. The chronic fatigue and other persistent symptoms many patients experience could result from continuous disruption of cellular energy metabolism and ongoing inflammation caused by the virus’s attack on mitochondria [38,41]

In summary, the structure of the SARS-CoV-2 E protein, which acts as a pentameric ion channel, is directly linked to its toxic effects. By disrupting normal interactions between the ER and mitochondria, the E protein mainly interferes with calcium regulation within cells, leading to severe mitochondrial dysfunction, oxidative stress, and excessive inflammation. From a therapeutic standpoint, targeting the E protein’s ion channel activity or developing treatments to stabilize mitochondrial function could help decrease the serious clinical outcomes of this disease. 

SARS-CoV-2 Non-Structural Proteins (NSPs) and mitochondria: their structural and functional relationships with clinical implications. The non-structural proteins (NSPs) of SARS-CoV-2 disrupt several critical cellular functions. One key target of this viral interference is the mitochondrion, which not only generates energy for the cell but also plays a crucial role in the innate immune response. 

Different NSPs target mitochondria to suppress antiviral defenses, disrupt energy metabolism, and promote a pro-viral environment in cells [46,47]. 

Notably, the virus’s replication and transcription complex (RTC), composed of NSPs, plays a crucial role in damaging mitochondria. This highlights the importance of regulating mitochondria for efficient viral replication [46,47].

Several NSPs have been identified as key players in this toxic interaction between the virus and mitochondria, each using different methods based on their unique structures. Specifically:

Nsp2 and Nsp4 produce double-membrane vesicles (DMVs) that act as organelles for viral replication and originate from the endoplasmic reticulum (ER). Nsp4 is a transmembrane protein that causes membrane curvature, while Nsp2 interacts with host proteins involved in transporting substances within the cell. Their functions enable the viral replication machinery to approach the ER–mitochondria contact sites. This proximity allows the virus to extract cellular lipids and ATP from the mitochondria, providing energy and resources to build its replication factories. This activity damages the mitochondria, impairing their function and causing energy failure [47,48].

Nsp5 (Main Protease, Mpro) plays a crucial role in cleaving the viral polyprotein into functional NSPs. Additionally, Nsp5 can target host proteins involved in essential cellular pathways, including those related to antiviral immunity. Specifically, Nsp5 cleaves key components of the mitochondrial antiviral-signaling (MAVS) pathway, impairing the cell’s primary defense against RNA virus infection. This targeted cleavage prevents the activation of type I interferons, which are the body’s most powerful antiviral signals [46,49].

Nsp12 (RNA-dependent RNA polymerase, RdRp) and its cofactors (Nsp7, Nsp8) act as the “energy thieves.” Nsp12 is the primary enzyme responsible for viral genome replication and forms a complex with its cofactors Nsp7 and Nsp8. Interestingly, this complex is located near the mitochondrial outer membrane because RNA synthesis requires a high supply of ATP and nucleotide triphosphates (NTPs). This placement ensures the virus has quick access to these essential resources, ultimately draining the cell’s energy and causing mitochondrial stress [47,50].

Nsp13 is a viral helicase essential for unwinding the viral RNA genome. Additionally, it has nucleotide triphosphatase (NTPase) activity. Furthermore, Nsp13 disrupts mitochondrial protein import and function, blocking the normal flow of proteins into the mitochondria. This way, Nsp13 hampers the organelle’s ability to perform its vital functions, including its role in antiviral defense [47,51].

The systematic disruption of mitochondrial function caused by SARS-CoV-2 NSPs has serious clinical consequences that greatly contribute to COVID-19 pathology.

Suppression of innate immunity: The primary clinical effect is the significant immunosuppression caused by proteins like Nsp5. By inhibiting the MAVS pathway, the virus delays and weakens the initial interferon response. This allows the virus to replicate freely during the early stages of infection, resulting in a higher viral load and more severe illness [47,52].

Fueling the cytokine storm: While the initial immune response is mild, significant mitochondrial damage and cell death (apoptosis) release danger-associated molecular patterns (DAMPs), such as mitochondrial DNA and reactive oxygen species (ROS). This cellular debris eventually triggers a delayed, yet exaggerated and dysfunctional, inflammatory response. The resulting “cytokine storm,” characterized by a massive release of pro-inflammatory cytokines, is a hallmark of severe COVID-19 and leads to acute respiratory distress syndrome (ARDS) and multi-organ failure [47,53].

Bioenergetic failure and organ damage: Reduction in ATP and mitochondrial dysfunction causes a severe cellular energy crisis. This damage is especially detrimental to organ systems that rely heavily on energy, such as the heart, kidneys, and brain. Mitochondrial injury directly results in cardiac damage (myocarditis), acute kidney injury, and neurological symptoms like “brain fog” in COVID-19 patients [46,47,54].

Importantly, in Long COVID (PASC), ongoing damage to the mitochondrial network is a leading theory explaining the persistent symptoms experienced by individuals with the typical Post-Acute Sequelae of COVID-19. Interestingly, symptoms like extreme fatigue, muscle weakness, and cognitive issues closely resemble what is seen with chronic mitochondrial dysfunction and ongoing cellular energy depletion [55].

In summary, the non-structural proteins of SARS-CoV-2 play a vital pathogenic role in the virus’s replication process through a coordinated and redundant strategy. NSPs interact with mitochondria at various levels, suppress innate immunity, exploit cellular resources, and trigger a series of events that ultimately lead to severe COVID-19 outcomes. Understanding these complex molecular interactions could be crucial for developing targeted therapies that protect mitochondria and restore immune function in patients affected by these conditions [51]. Additionally, this knowledge could help clarify some unclear pathogenetic mechanisms underlying other diseases, such as sepsis.

Clinical implications: The structural and functional disruptions in mitochondria caused by SARS-CoV-2 NSPs have important clinical implications, affecting the development and severity of COVID-19.

Multi-organ dysfunction: Since mitochondria are found throughout the body, their dysfunction can affect nearly every organ system. This explains the multi-organ issues seen in severe COVID-19, such as respiratory failure, heart injury, kidney damage, and neurological symptoms [56].

Long COVID: Persistent mitochondrial dysfunction and energy deficits are believed to contribute to ongoing fatigue and other severe symptoms experienced by individuals with Long COVID. The inability of cells to produce enough ATP can explain prolonged exhaustion [55].

Inflammatory storm and tissue damage: The increased ROS production and dysregulated immune responses caused by mitochondrial stress lead to systemic inflammation, endothelial dysfunction, and widespread tissue damage in severe cases [35].

Thrombosis: Mitochondrial dysfunction can also promote a procoagulant state, thereby increasing the risk of thrombosis and microvascular clots, a common complication associated with COVID-19 [35,57].

Neurological manifestations: Brain cells rely heavily on mitochondrial function. NSP-induced mitochondrial damage can cause neurological symptoms such as brain fog; headaches; and, in severe cases, encephalopathy [35,58].

## 2. SARS-CoV-2 Accessory Proteins (ORFs) and Mitochondria: Structural and Functional Relationships with Clinical Implications

SARS-CoV-2 also encodes several accessory proteins, primarily designated as ORFx. These proteins are not always essential for replication in cell culture, but they play important roles in viral pathogenesis and in interacting with the host immune system. This group of accessory proteins interacts strongly with host cellular machinery, including mitochondria. By causing mitochondrial dysfunction, these proteins support viral replication, evade host immune defenses, and ultimately damage mitochondria. They also contribute to the development and diversity of clinical features of COVID-19 [59,60]. 

### 2.1. ORF3a

ORF3a functions as a viroporin, forming ion channels in various cellular membranes, including the mitochondria. Specifically, it interacts with the mitochondrial ATP-sensitive potassium channel (MitoK_ATP). Modulating this channel can lead to mitochondrial disruption through multiple mechanisms [61].

Disruption of the electrochemical gradient required for ATP production, along with increased Reactive Oxygen Species (ROS), leads to oxidative stress and cell damage. This triggers mitochondrial-mediated apoptosis. Calcium balance becomes unregulated. From a disease standpoint, ORF3a-induced apoptosis and inflammatory responses, driven by the interferon-β signaling pathway, are essential. Also, its viroporin activity further contributes to lung damage and immune system problems [59,61].

### 2.2. ORF3c

ORF3c is a small, less-studied protein primarily involved in disrupting host immune defenses. Its harmful effects depend on [62,63]:

i. Manipulation of mitochondrial fission and fusion (alteration of normal mitochondrial dynamics).
ii. MAVS degradation (suppressing the host’s innate antiviral response).
iii. Disruption of mitochondrial bioenergetics: interfering with respiration, ATP production, or other metabolic pathways to enhance viral replication.
iv. Dysregulation of calcium homeostasis.
v. Activation of cell death pathways, like apoptosis or necroptosis.

The clinical implications of these hypothesized interactions include increased inflammation and cytokine storms, which can cause widespread cellular damage and organ failure, such as in the lungs, heart, or kidneys. This may worsen severe COVID-19 and contribute to Post-Acute Sequelae of SARS-CoV-2 Infection (PASC) or Long COVID [59,64].

### 2.3. ORF6

ORF6 primarily antagonizes interferon signaling by blocking the nuclear import of interferon. However, evidence suggests its involvement in mitochondrial stress. 

Studies using fluorescence microscopy have shown that ORF6 is located near or within mitochondria in infected cells.

Potential pathogenic roles of mitochondria include:

Mitochondrial dynamics: ORF6 may affect fusion and fission, resulting in fragmentation or altered structure, which can impair respiration and ATP production.

Interference with Mitochondrial Antiviral Signaling (MAVS): Although the direct interaction of ORF6 with MAVS is under investigation, its general role in dampening antiviral responses may indirectly impact mitochondrial immune functions.

Bioenergetic impairment: If ORF6 directly or indirectly impacts mitochondrial bioenergetics, it could contribute to cellular damage and inflammation in severe COVID-19.

Clinically, if ORF6 disrupts mitochondrial homeostasis, it could exacerbate inflammation, contribute to cell death in various tissues (e.g., the lungs, heart, and brain), and worsen the overall pathology of COVID-19 [59,64].

### 2.4. ORF7a

This accessory protein plays a role in immune evasion and apoptosis. It can trigger cell death, possibly through mitochondrial pathways. ORF7a has also been shown to interfere with host antiviral responses, which may rely on disrupted mitochondrial-dependent signaling pathways [59,65].

### 2.5. ORF7b

This is a small (43-amino-acid) transmembrane protein with crucial structural and functional interactions, indirectly impacting mitochondria. Specifically, ORF7b features a hydrophobic transmembrane segment that creates a leucine zipper motif, supporting multimerization and membrane anchoring, and may also function as a viroporin, which can weaken membrane integrity.

Although direct mitochondrial localization has not yet been confirmed, ORF7b’s interaction with over 1700 human proteins—especially those involved in ER-Golgi transport, glucose metabolism, and immune signaling—suggests indirect effects on mitochondria, such as:

Disruption of MAVS: Similar to ORF9b, possibly suppressing antiviral responses.

Modulation of apoptosis pathways: Influencing cell survival or death.

ER stress induction: Closely linked to mitochondrial dysfunction, impacting cellular homeostasis.

Suppression of Interferon (IFN) production: Aiding viral immune evasion.

The promotion of pro-inflammatory cytokines like IFN-β, TNF-α, and IL-6 plays a role in the cytokine storm observed in severe COVID-19 [59,66].

### 2.6. ORF8

ORF8 is a 121-amino acid accessory protein with an Ig-like fold, a signal peptide for ER import, and a unique ability to form dimers through disulfide bonds. It is a highly variable protein with frequent mutations and deletions (mutations or deletions in ORF8—e.g., Δ382—generally correlate with reduced inflammation and better clinical outcomes). It may modulate immune responses, promoting viral evasion. It has been linked to the unfolded protein response and endoplasmic reticulum (ER) stress. Notably, due to the close relationship between the ER and mitochondria, ER stress induced by ORF8 can indirectly impact mitochondrial function, thereby contributing to cellular dysfunction. Additionally, studies have shown that ORF8 can suppress MHC-I expression via autophagy-dependent lysosomal degradation, impairing antigen presentation. It also activates the IL-17 and IFN-γ pathways, contributing to cytokine storms and immune dysregulation. Lastly, ORF8 signals through MyD88 in macrophages and monocytes, bypassing IL-17 receptors. From a clinical perspective, ORF8 contributes to increased lung inflammation and macrophage infiltration, worsening disease progression [67,68]. 

### 2.7. ORF9b

ORF9b is a well-characterized accessory protein that directly targets mitochondria. It localizes to the outer mitochondrial membrane (OMM) and induces mitochondrial fragmentation. A key interaction involves ORF9b binding to TOM70, a component of the translocase of the outer membrane (TOM) complex, which is crucial for: (i) importing mitochondrial precursor proteins, and (ii) mediating antiviral signaling via MAVS. Specifically, ORF9b binds to the C-terminal domain of TOM70, occupying a hydrophobic pocket. This binding disrupts TOM70’s interaction with Hsp90 and MAVS, thereby suppressing type I interferon responses. Pathogenetically, by impairing TOM70-mediated import of mitochondrial proteins, ORF9b leads to: • depletion of mitochondrial proteins, • reduction in mitochondrial volume, and • disruption of mitochondrial biogenesis. Moreover, ORF9b-dependent mitochondrial damage and the subsequent release of mitochondrial DNA (mtDNA) can activate pro-inflammatory pathways (e.g., cGAS/STING, NLRP3 inflammasome), contributing to apoptosis and cytokine storms observed in severe COVID-19 cases. Clinically, this suppression contributes to immune evasion and may exacerbate disease severity, particularly in elderly individuals with compromised mitochondrial function [59,69]. 

### 2.8. ORF9c

While less extensively studied than other ORFs, emerging evidence suggests that SARS-CoV-2 ORF9c interacts with host cellular processes that could affect mitochondrial function. It seems to interact with mitochondrial proteins involved in electron transport and immune signaling, including ECSIT, ACAD9, and NLRX1.

Importantly, ORF9c localizes to mitochondria and disrupts their cristae structure, which is essential for ATP production. Additionally, it alters the expression of genes related to amino acid metabolism, oxidative phosphorylation, and immune regulation (by suppressing antiviral responses, particularly interferon signaling), and promotes inflammatory signaling via NLRP3 activation.

From a clinical perspective, this protein helps with immune evasion, viral persistence, and hyperinflammation [59,70].

### 2.9. ORF10

ORF10 is a small viral protein (38 amino acids) unique to SARS-CoV-2. Its effects on mitochondria include:

- Binding to mitochondrial proteins: Interacting with proteins like NIX and LC3B to promote mitophagy—the selective removal of damaged mitochondria.
- Immune suppression: breaking down MAVS to prevent interferon responses.
- Metabolic effects: Disrupting amino acid metabolism and aiding in cellular reprogramming [59,71].

In conclusion, these accessory proteins play a vital role in the physiopathology of COVID, especially across various organs and systems, such as:

Lung: Mitochondrial dysfunction in lung epithelial cells and immune cells can worsen acute respiratory distress syndrome (ARDS) and lung fibrosis.

Heart: Cardiac complications, such as myocarditis and arrhythmias, can be associated with mitochondrial damage in cardiomyocytes.

Brain: Neurological symptoms, such as neuroinflammation and cognitive impairment, may be related to mitochondrial dysfunction in neuronal and glial cells.

### 2.10. Potential Therapeutic Strategies

Understanding these pathogenetic molecular mechanisms can help in developing therapeutic interventions. Here are some examples (Table 2):

Mitochondrial-targeted antioxidants: These compounds help reduce ROS production and oxidative stress.

Mitochondrial biogenesis enhancers: drugs that stimulate the production of new, healthy mitochondria could help restore cellular energy balance.

Modulators of mitochondrial dynamics: therapies that target the balance between mitochondrial fission and fusion could enhance mitochondrial health.

Targeting NSP–mitochondrial interactions: specific inhibitors designed to prevent NSPs from binding to mitochondrial proteins could stop subsequent mitochondrial damage.

In conclusion, the SARS-CoV-2 non-structural proteins exhibit a close interaction with host mitochondria, resulting in significant structural and functional alterations. As a result, these proteins disrupt mitochondria and play a key role in the development of COVID-19, leading to multi-organ failure, inflammation, and typical severe symptoms. 

## 3. Conclusions

In summary, an intriguing aspect of the physiopathogenesis of SARS-CoV-2 comes from its unique protein framework, which interacts redundantly with mitochondria (i.e., different viral proteins target the same mitochondrial function). These complex damage pathways directly cause: 

Disruption of mitochondrial protein import and function caused by a direct interaction between mitochondrial proteins (TOM70) and ORF9b/ORF9c.

Impairment of mitochondrial bioenergetics: virus proteins directly damage ETC components, and altered expression of genes involved in mitochondrial respiration causes a reduction in cellular energy production.

Increased oxidative stress: Virus-damaged ETC boosts reactive oxygen species (ROS) production.

Disruption of mitochondrial dynamics: SARS-CoV-2 disturbs the balance of mitochondrial fusion and fission, resulting in fragmented and dysfunctional mitochondria.

Release of Mitochondrial DAMPs (mtDAMPs): in the form of mitochondrial DNA (mtDNA)—unmethylated CpG motifs in mtDNA resemble bacterial DNA and act as potent activators of immune cells (e.g., via TLR9), leading to inflammation. mtDNA can also directly activate the coagulation cascade and induce NET formation; cardiolipin—when exposed, can act as an autoantigen and contribute to antiphospholipid syndrome-like phenomena, promoting thrombosis; N-formyl peptides—mitochondrial proteins with N-formyl methionine at their N-terminus are potent chemoattractants for neutrophils and can activate inflammatory responses.

Excessive immune response (i.e., cytokine storm) that further causes mitochondrial damage and cellular stress.

### Induction of Mitochondrial Apoptosis

**Figure 2:**
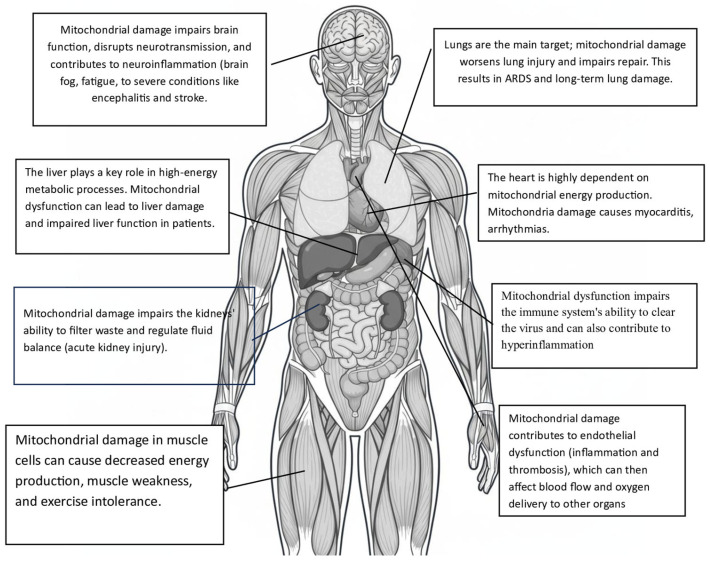
Organs and tissues affected secondarily by mitochondrial damage caused by SARS-CoV-2.

All these molecular abnormalities at the organ and tissue levels (i.e., mtDAMPs) lead to endothelial activation, which triggers the expression of tissue factor and adhesion molecules, thereby promoting coagulation and recruitment of leukocytes. mtDAMPs can also directly activate platelets, resulting in platelet aggregation (Figure 2). Additionally, these same changes strongly induce NETs, which are prothrombotic scaffolds made of DNA and granular proteins. They also activate complement and coagulation cascades, initiating and propagating DIC. Similarly, these mechanisms appear to contribute to other common COVID-19 complications, such as metabolic disorders, kidney failure, encephalopathy, cardiovascular problems (e.g., ischemia, shock), and sepsis.

From all this, the central role of mitochondrial damage in COVID’s pathophysiology is clear. Equally important is the evidence that mitochondria play a significant, yet still underestimated, pathogenic role in various diseases. 

A more precise understanding of the molecular mechanisms behind these mitochondriopathies can significantly impact diagnosis, prognosis, and especially treatment in various essential and current human diseases.

## Figures and Tables

**Table 1:** SARS-CoV-2 proteome and mitochondria: structural and functional relationships.

SARS-CoV-2 Protein	Type (Structural/Non-Structural/Accessory)	Primary Role in Virus	Mitochondrial Interaction/Impact
ORF9b	Accessory Protein	Unknown direct viral function; known host interaction	Targets outer mitochondrial membrane (OMM); induces mitochondrial fragmentation; inhibits mitochondrial protein import; impairs mitochondrial dynamics and function.
Nsp5 (3CLpro)	Non-Structural Protein	Main protease; cleaves viral polyprotein	Indirectly impacts mitochondria by increasing cellular stress due to essential role in viral replication.
Nsp12 (RdRp)	Non-Structural Protein	Core enzyme for viral genome replication and transcription	Places strain on mitochondrial ATP production due to high energy demands of viral replication.
Spike (S) protein	Structural Protein	Host cell entry	Induces inflammation and cellular stress; indirectly affects mitochondrial function in various tissues through systemic inflammation.
Envelope (E) protein	Structural Protein	Viral assembly and budding	Viroporin activity; alters ion homeostasis; potentially impacts mitochondrial calcium signaling and membrane potential.
Membrane (M) protein	Structural Protein	Viral assembly	Indirectly influences cellular processes relying on mitochondrial function through interactions with other viral proteins and host membranes.
Nucleocapsid (N) protein	Structural Protein	Binds viral RNA; replication and transcription	Contributes to cellular stress upon accumulation in the cytoplasm, which in turn affects mitochondria.

**Table 2:** SARS-CoV-2 and Mitochondria: Potential Therapeutic Opportunities.

Therapeutic Strategy	Rationale/Goal	Examples/Mechanism (Based on Provided Text)
Mitochondria-Targeted Antioxidants	To combat oxidative stress induced by SARS-CoV-2 infection.	Focus on neutralizing Reactive Oxygen Species (ROS) within mitochondria.
Mitochondrial Biogenesis Enhancers	To promote the formation of new, healthy mitochondria.	Aim to increase the number and quality of mitochondria within cells.
Drugs Modulating Mitochondrial Dynamics	To restore proper balance between mitochondrial fission and fusion.	Address issues like excessive mitochondrial fragmentation or impaired fusion.
Inhibitors of Viral Proteins Targeting Mitochondria	To prevent mitochondrial damage caused by specific viral proteins.	Specifically target proteins like ORF9b to block their detrimental effects on mitochondria.
Metabolic Reprogramming Agents	To support overall mitochondrial function and cellular metabolism.	Aims to optimize metabolic pathways that rely on or are linked to mitochondria.

## Data Availability

No new data were created or analyzed in this study. Data sharing is not applicable to this article.
